# Impaired Stomatal Control Is Associated with Reduced Photosynthetic Physiology in Crop Species Grown at Elevated [CO_2_]

**DOI:** 10.3389/fpls.2016.01568

**Published:** 2016-10-25

**Authors:** Matthew Haworth, Dilek Killi, Alessandro Materassi, Antonio Raschi, Mauro Centritto

**Affiliations:** ^1^National Research Council – Tree and Timber InstituteFlorence, Italy; ^2^Department of Agrifood Production and Environmental Sciences, University of FlorenceFlorence, Italy; ^3^National Research Council – Institute of BiometeorologyFlorence, Italy

**Keywords:** stomatal behavior, stomatal evolution, stomatal sensitivity, drought, photosynthetic down-regulation, food security

## Abstract

Physiological control of stomatal conductance (*G*_s_) permits plants to balance CO_2_-uptake for photosynthesis (*P*_N_) against water-loss, so optimizing water use efficiency (WUE). An increase in the atmospheric concentration of carbon dioxide ([CO_2_]) will result in a stimulation of *P*_N_ and reduction of *G*_s_ in many plants, enhancing carbon gain while reducing water-loss. It has also been hypothesized that the increase in WUE associated with lower *G*_s_ at elevated [CO_2_] would reduce the negative impacts of drought on many crops. Despite the large number of CO_2_-enrichment studies to date, there is relatively little information regarding the effect of elevated [CO_2_] on stomatal control. Five crop species with active physiological stomatal behavior were grown at ambient (400 ppm) and elevated (2000 ppm) [CO_2_]. We investigated the relationship between stomatal function, stomatal size, and photosynthetic capacity in the five species, and then assessed the mechanistic effect of elevated [CO_2_] on photosynthetic physiology, stomatal sensitivity to [CO_2_] and the effectiveness of stomatal closure to darkness. We observed positive relationships between the speed of stomatal response and the maximum rates of *P*_N_ and *G*_s_ sustained by the plants; indicative of close co-ordination of stomatal behavior and *P*_N_. In contrast to previous studies we did not observe a negative relationship between speed of stomatal response and stomatal size. The sensitivity of stomata to [CO_2_] declined with the ribulose-1,5-bisphosphate limited rate of *P*_N_ at elevated [CO_2_]. The effectiveness of stomatal closure was also impaired at high [CO_2_]. Growth at elevated [CO_2_] did not affect the performance of photosystem II indicating that high [CO_2_] had not induced damage to the photosynthetic physiology, and suggesting that photosynthetic control of *G*_s_ is either directly impaired at high [CO_2_], sensing/signaling of environmental change is disrupted or elevated [CO_2_] causes some physical effect that constrains stomatal opening/closing. This study indicates that while elevated [CO_2_] may improve the WUE of crops under normal growth conditions, impaired stomatal control may increase the vulnerability of plants to water deficit and high temperatures.

## Introduction

Stomatal pores act as the interface between the plant and the atmosphere, regulating the uptake of CO_2_ for photosynthesis (*P*_N_) and the loss of water via transpiration. Photosynthesis rates are positively related to stomatal conductance (*G*_s_; Wong et al., 1979; Flexas and Medrano, 2002), and effective stomatal control through morphological changes to the number of stomata and physiological regulation of stomatal aperture size allows the optimal balance of CO_2_-uptake and water-loss over a range of favorable and sub-optimal growth conditions (Flexas et al., 2002; Lauteri et al., 2014; Haworth et al., 2015). Physiological regulation of the size of the stomatal pore aperture ranges from ‘active’ to ‘passive’ stomatal behavior. Active stomatal behavior involves rapid alteration of stomatal aperture following an external stimulus as ions are actively pumped across guard cell membranes to alter guard cell turgor. Alternatively, where guard cell turgor, and as a result pore area, follows whole leaf turgor, stomatal behavior is considered to be passive (Chater et al., 2013). The development of crop species has involved the selection of more productive and faster growing varieties over multiple generations (Evans, 1980; Roche, 2015). These selected varieties often possess greater leaf area rates of *P*_N_ than their less productive counterparts (Zelitch, 1982; Fischer et al., 1998; Gu et al., 2014). As a result, the vast majority of crops currently cultivated possess active stomatal physiological behavior that permits the levels of *G*_s_ required to sustain high *P*_N_, but also the capacity to respond rapidly to a change in environmental conditions (Kalaji and Nalborczyk, 1991; Haworth et al., 2015; Roche, 2015). However, it is unclear how active physiological stomatal behavior in crop plants can be affected by changes in the atmospheric concentration of [CO_2_], and whether growth at elevated [CO_2_] can induce a loss of stomatal control.

Rising atmospheric [CO_2_] is considered to have a beneficial effect on the carbon balance of plants though CO_2_-fertilization and reduced transpirative water-loss generally associated with lower *G*_s_ that results in an increase in water use efficiency (WUE; Centritto et al., 2002; Wullschleger et al., 2002; Haworth et al., 2016). Nonetheless, climate change will also result in an increase in the frequency, severity and duration of drought, and increased temperature events (Ciais et al., 2005). In these cases, effective stomatal control is crucial to the plant stress response (e.g., Bunce, 2000; Shah and Paulsen, 2003; Killi et al., 2016), particularly in fast-growing crop species with high maximum rates of *G*_s_. It has been suggested that smaller stomata are able to open and close more rapidly, thus affording greater responsiveness to a change in growth conditions (Hetherington and Woodward, 2003; Drake et al., 2013). Plants with large numbers of small stomata are also able to maintain greater levels of *G*_s_ (de Boer et al., 2016), potentially as an adaptation to declining [CO_2_] over the Cenozoic (Franks and Beerling, 2009). It may therefore be expected that stomatal size, the speed of stomatal closure and maximal rates of *P*_N_ and *G*_s_ are closely associated in fast-growing plants with active stomatal behavior. Nevertheless, the impact of growth at elevated [CO_2_] on the regulation of stomatal aperture is largely unknown.

Stomatal aperture is regulated by network of interlinked signals (Hetherington and Woodward, 2003) incorporating photosynthesis in the light (Messinger et al., 2006) and the physiological status of the plant (e.g., Lauteri et al., 2014). Beech (*Fagus sylvatica*), chestnut (*Castanea sativa*), and oak (*Quercus robur*) all exhibit an inverse relationship between *G*_s_ and leaf to air vapor pressure deficit (VPD) under light conditions when grown at ambient [CO_2_]. However, when grown in atmospheres enriched in [CO_2_] to 710 ppm, oak and chestnut showed reduced stomatal sensitivity to VPD, while beech no longer altered *G*_s_ to VPD (Heath, 1998). Growth at elevated [CO_2_] did not induce a reduction of *G*_s_ in beech, but also reduced the speed (∼-25% after 4 days) and tightness (∼-22% over a soil water potential range of -200 to -250 hPa) of stomatal closure in response to soil drying. This impaired stomatal control was associated with reduced stomatal sensitivity to the drought stress hormone ABA in the beech plants grown at elevated [CO_2_]. Furthermore, an increase in leaf area, alongside the loss of stomatal control incurred at high [CO_2_], made the beech trees more susceptible to drought stress when grown in atmospheres enriched in [CO_2_] (Heath and Kerstiens, 1997). The cycad *Lepidozamia peroffskyana* and broad-leaved conifer *Nageia nagi* both exhibit active stomatal control when grown at ambient [CO_2_] under well-watered conditions. However, when grown at elevated [CO_2_] of 1500 ppm, both species no longer adjusted *G*_s_ in response to an instantaneous change in external [CO_2_] (*C*_a_; Haworth et al., 2013), indicative of a loss of stomatal control. Hollyfern (*Cyrtomium fortunei*) also showed a loss of stomatal sensitivity to *C*_a_ and impaired stomatal closure during darkness when grown in atmospheres of 2000 ppm [CO_2_] (Haworth et al., 2015). Such a loss of stomatal control at high [CO_2_] would impair the capacity of plants to limit water-loss associated with *P*_N_ during episodes of high transpirative demand.

Effective stomatal control is not only important during light-driven assimilation of CO_2_, but also when conditions are not favorable for *P*_N_ (Killi et al., 2016). At night many plants do not close their stomata to their full extent, resulting in transpirative water-loss in the absence of *P*_N_. Night-time *G*_s_ in Eucalyptus (*Eucalyptus sideroxylon*) is 30% of levels recorded in the day-time (Zeppel et al., 2012), and can be as high as 250 mmol m^-2^ s^-1^ in many plants (Caird et al., 2007), thus representing a significant loss of water. The ecological function of night-time *G*_s_ is unclear, but is hypothesized to be related to the maintenance of root mass-flow of water to ensure the uptake of mobile nutrients such as nitrogen (Caird et al., 2007). Under elevated [CO_2_] of 640 ppm and not experiencing drought, Eucalyptus exhibited a 17% increase in night-time *G*_s_, but levels of *G*_s_ during the day-time were identical at ambient and elevated [CO_2_] (Zeppel et al., 2012); possibly as an adaptation to enhance nutrient uptake due to reduced root mass-flow of water under elevated [CO_2_] (Van Vuuren et al., 1997). Under optimal growth conditions, during the night plant water potential generally equilibrates with that of soil water potential (Hinckley et al., 1978; Donovan et al., 2001). Increased night-time *G*_s_ may impair the equilibration of leaf and soil water potentials, or in the case of promoting root mass-flow to enhance nutrient uptake may indicate some underlying nutrient deficiency in the plants. This increase in night-time *G*_s_ under elevated [CO_2_] may seem somewhat incongruous, as an increase in [CO_2_] is widely considered likely to reduce *G*_s_ (Centritto et al., 1999; Ainsworth and Rogers, 2007). However, a rise in [CO_2_] may impair stomatal function, resulting in stomatal pores that close more slowly and less tightly (e.g., Haworth et al., 2015), potentially accounting for observations of increased night-time *G*_s_ at elevated [CO_2_] (Zeppel et al., 2012). Measurement of the speed of the stomatal response of plants grown under elevated [CO_2_] to darkness (i.e., conditions no longer conducive to *P*_N_) and the tightness of stomatal closure (Haworth et al., 2015) may provide indications of any loss of stomatal function associated with high [CO_2_].

The loss of stomatal control represented by the reduced capacity and speed of stomatal closure at elevated [CO_2_] may render plants vulnerable to desiccation during episodes of drought or high transpirative demand. This is counter to the prevailing consensus that an increase in [CO_2_] would improve WUE (Centritto et al., 2002; Ainsworth and Rogers, 2007) and mitigate drought by reducing water-loss (Wall, 2001). A loss of stomatal control and increased vulnerability to drought and heat-waves would have severe implications for crops such as wheat (e.g., Stratonovitch and Semenov, 2015). However, there is comparatively little information regarding the impact of elevated [CO_2_] on stomatal control in crop species. Photosynthesis in the mesophyll layer and *G*_s_ are closely co-ordinated via *C*_i_ in the presence of red light (Messinger et al., 2006; Engineer et al., 2016). It is possible that any effect of elevated [CO_2_] may have an effect upon the regulation of stomatal aperture via a direct effect on *P*_N_. To study the mechanistic effects of elevated [CO_2_] on stomatal control in C3 crop plants we grew oat (*Avena sativa*), sunflower (*Helianthus annuus*), cotton (*Gossypium hirsutum*), barley (*Hordeum vulgare*), and wheat (*Triticum aestivum*) under ambient (400 ppm) and elevated (2000 ppm) [CO_2_]. As comparatively little is known about how growth at elevated [CO_2_] can affect stomatal control, a higher [CO_2_] level was chosen for the elevated treatment than concentrations predicted by the IPCC (2007) models, but not in the context of atmospheric [CO_2_] over the last 200 million years (Berner, 2009), as this would clearly demonstrate whether growth at elevated [CO_2_] has a mechanistic effect on stomatal function. The effect of growth at elevated [CO_2_] on stomatal control (tightness of closure following a cessation of illumination and sensitivity to instantaneous changes in *C*_a_: reported in a previous study, with the exception of wheat, of Haworth et al., 2015) and the interaction with photosynthetic physiology were investigated. We hypothesize that these fast-growing crop species with high rates of *P*_N_ will exhibit a high degree of stomatal control, but that this stomatal control may become impaired at elevated [CO_2_]. This study aims to: (i) investigate potential correlations between stomatal pore size and the speed of stomatal closure, and whether species with ‘fast’ stomata possess greater maximum rates of *G*_s_ and *P*_N_; (ii) determine how stomatal control in crop plants with active physiological stomatal behavior is affected by growth at elevated [CO_2_] through analysis of speed and tightness of stomatal closure following the cessation of illumination and stomatal sensitivity to instantaneous increases in *C*_a_; (iii) assess whether adjustment of the photosynthetic physiology may affect stomatal control at high [CO_2_] either through alteration of the ‘photosynthetic’ stomatal response to [CO_2_], or damage to the photosynthetic apparatus incurred by growth at elevated [CO_2_], and; (iv) explore the possible implications of any change in stomatal control for crop plants growing in a future high [CO_2_] and water-limited world.

## Materials and Methods

### Plant Growth Conditions

The plants were potted in 6 dm^3^ square pots using a 5:1 mixture of commercial compost and vermiculite and placed in two large walk-in growth rooms with full control of light, temperature, [CO_2_] (one chamber maintained ambient atmospheric [CO_2_] of 400 ppm and the second an elevated [CO_2_] level of 2000 ppm) and humidity for 16-week (technical details of the plant growth chambers are given in Materassi et al., 2005). The plants were watered to pot capacity every 2 days and provided weekly with a commercial liquid plant fertilizer (COMPO Concime Universale, NPK 7-5-7, B, Cu, Fe, Mn, Mo, Zn: COMPO Italia, Cesano Maderno, Italy) to facilitate nutrient availability at free access rates. The growth chambers maintained conditions of 16 h of daylight (14 h at full *PAR* levels of 1000 μmol m^-2^ s^-1^ with two 1-h periods of simulated dawn/dusk where light intensity was incrementally increased/decreased), a day and night-time temperature regime of 25/18°C and constant relative humidity of 50%. To avoid potential chamber effects, the growth rooms were alternated every 2 weeks – no significant differences were observed in the measurements conducted under the same conditions in different growth chambers. Timings of day/night programs on the plant growth chambers were staggered to allow the maximum number of plants to be analyzed at the optimal time of the day/night program for photosynthetic activity (07:00–11:00 h), and thus avoid the influence of circadian stomatal behavior; particularly where stomata close at midday or during the early afternoon when leaf water potentials may decrease. Four replicates of each species were grown under ambient and elevated [CO_2_], and a minimum of three replicates were used for the measurement of stomatal sensitivity to *C*_a_ and closure in response to darkness.

### Leaf Gas-Exchange Analysis of Stomatal Control

**FIGURE 1:**
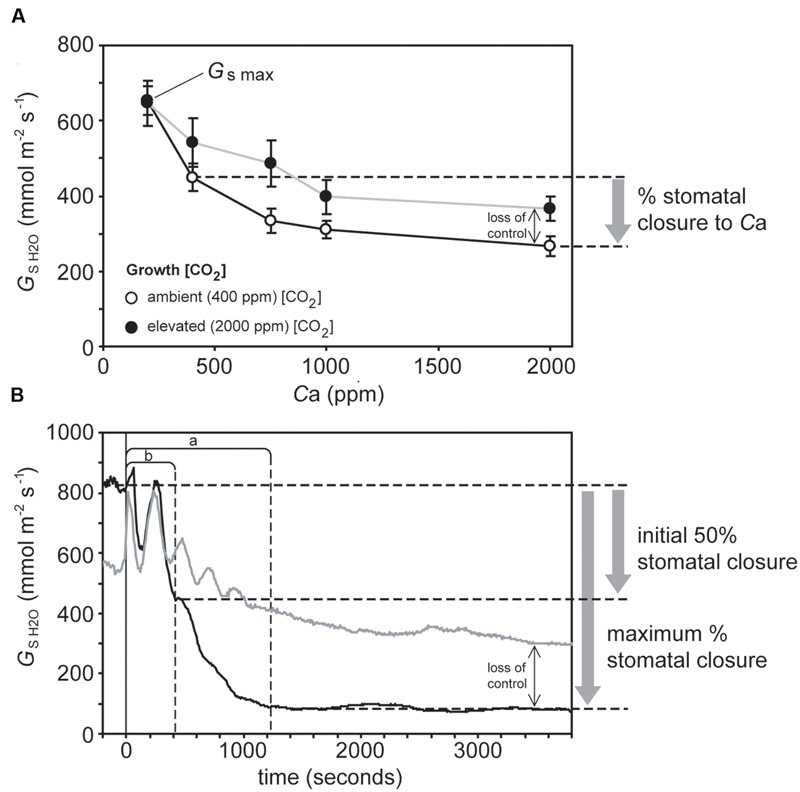
**Illustration of gas exchange measurements in sunflower to determine: **(A)** Stomatal sensitivity to *C*_a_ – plants grown in atmospheres of 400 ppm (open symbols, black line) and 2000 ppm (closed symbols, gray line) were exposed to instantaneous increases in *C*_a_.** Stomatal conductance was allowed to stabilize at each *C*_a_ before data was logged. The difference between *G*_s_ values at *C*_a_ values of 400 and 2000 ppm [CO_2_] is used to infer stomatal closure. **(B)** Stomatal closure to darkness – the leaves of plants grown in atmospheres of 400 ppm (black line) and 2000 ppm (gray line) were placed in a cuvette, after *G*_s_ had remained stable for 10 min the light in the cuvette and room were simultaneously switched off (represented by the vertical black line). Fifty percent of the maximum and maximum stomatal closure are marked by dashed horizontal lines. The speed of stomatal closure at 50% and maximum stomatal closure are marked by dashed vertical lines labeled *a* and *b*, respectively. The lack of stability in *G*_s_ values immediately after the cessation of illumination may be an artifact related to changes in the temperature of the leaf cuvette affecting the measurement of relative humidity of the air within the cuvette. For clarity, the points taken to illustrate the determination of stomatal closure are only showed in relation to the plants grown at 400 ppm [CO_2_] (black lines). Plants grown at 2000 ppm (gray lines) are included to illustrate the impact of [CO_2_] on stomatal control, with the black arrow indicating the loss of stomatal control incurred by growth at elevated [CO_2_].

A PP-Systems Ciras-2 attached to a PLC6(U) leaf cuvette and LED light unit (PP-Systems, Amesbury, MA, USA) was used to gauge the physiological response of stomata to darkness and *C*_a_. All physiological measurements were conducted on the same leaf per plant (no more than one measurement was performed on each plant over 48 h) in a well-ventilated air-conditioned room maintained at 25°C. The newest fully expanded leaf was consistently used for analysis (in grasses this was the leaf below the flag leaf) to avoid any age-related effects regarding stomatal functionality. To assess the physiological stomatal behavioral responses of the plants to *C*_a_, the level of [CO_2_] within the leaf cuvette was increased in a number of stages (200, 400, 750, 1000, and 2000 ppm CO_2_) while temperature (25°C), VPD (1.6–1.8 KPa ± 0.1 KPa), and light intensity (2000 μmol m^-2^ s^-1^) remained constant. At each [CO_2_] step, the stomatal conductance of water vapor (*G*_s H2O_) was allowed to stabilize and then remain stable for 5–10 min before being recorded. Maximum stomatal conductance (*G*_s max_) was considered to be *G*_s H2O_ recorded at a [CO_2_] of 50 ppm to induce full stomatal opening (Centritto et al., 2003). Stomatal closure was expressed as the percentage of *G*_s H2O_ values at 2000 ppm CO_2_ relative to those recorded at an ambient [CO_2_] of 400 ppm (**Figure 1**). To measure the stomatal response to darkness, a leaf was placed in the leaf cuvette with a temperature of 25°C, *PAR* of 2000 μmol m^-2^ s^-1^ and 400 ppm [CO_2_]. After *G*_s H2O_ had remained stable for 15 min the lights in both the cuvette and the room were simultaneously switched off and *G*_s H2O_ was recorded every 10 s for a minimum of 1 h (Meidner and Mansfield, 1965). Vapor pressure deficit in the cuvette was maintained between 1.6 to 1.8 KPa ± 0.1 KPa throughout the measurement of the stomatal response to darkness. Stomatal closure in response to the cessation of illumination was expressed as the percentage *G*_s H2O_ after 1-h of darkness. The speed of stomatal closure is not uniform after the onset of darkness (Haworth et al., 2015); the reduction in *G*_s H2O_ per second over the first 50% reduction in *G*_s H2O_, and also over the time to reach the maximum extent of stomatal closure was therefore determined (**Figure 1**). Measurements of stomatal pore length (SPL) of the plants under ambient and elevated [CO_2_] were taken from Haworth et al. (2015).

### Leaf Gas-Exchange and Chlorophyll Fluorescence Analysis of Photosynthetic Physiology

Response curves of *P*_N_ to increasing internal sub-stomatal [CO_2_] (*C*_i_) under a saturating light intensity of 2000 μmol m^-2^ s^-1^ (*C*_a_ sequence of 350, 250, 150, 50, 100, 200, 300, 400, 600, 800, 1000, 1200, 1400, 1600, 1800, and 2000 ppm) at a standard cuvette temperature of 25°C were recorded on the same leaves used to assess stomatal control using the same Ciras-2 photosynthesis system. The maximum carboxylation rate of RubisCO (*V*c_max_), the maximum rate of electron transport for regeneration of ribulose-1,5-bisphosphate (*J*_max_) and mesophyll conductance to CO_2_ (*G*_mCO2_) were calculated from the *P*_N_/*C*_i_ response curves following Ethier and Livingston (2004). The ‘curve fitting’ method of Ethier and Livingston (2004) estimates *G*_mCO2_ from the response of *P*_N_ to increasing partial pressure of CO_2_ in the sub-stomatal air-space assuming a constant *G*_mCO2_ across the range of *C*_i_ values (cf. Flexas et al., 2007). Total conductance to CO_2_ (*G*_totCO2_) was calculated as:

$$G_{\text{tot co2}}=\frac{\left(G_{\text{s co}2}*G_{\text{m co}2}\right)}{\left(G_{\text{s co}2}+G_{\text{m co}2}\right)}$$

Where *G*_sCO2_ is the stomatal conductance to CO_2_ – in the present manuscript to further aid differentiation between measurement of stomatal conductance to H_2_O and CO_2_, *G*_s H2O_ is expressed as mmol m^-2^ s^-1^, while *G*_s CO2_ is expressed as mol m^-2^ s^-1^. The maximum rate of photosynthesis (*P*_N max_) was considered to be *P*_N_ at a saturating light intensity of 2000 μmol m^-2^ s^-1^ and [CO_2_] of 2000 ppm. The maximum (*F*_v_/*F*_m_) and actual (ΦPSII: Δ*F*/*F*′m) quantum efficiency of photosystem II was recorded using a Hansatech FMS-2 (saturating pulse of 10,000 μmol m^-2^ s^-2^) and dark adaptation clips (Hansatech, King’s Lynn, UK) after 30 min of dark adaptation and exposure to actinic light of 1000 μmol m^-2^ s^-1^ for a minimum of 10 min after the first saturating pulse (Genty et al., 1989; Kalaji et al., 2014).

### Statistical Analyses

Statistical analyses were performed using SPSS 20 (IBM, New York, NY, USA). To test [CO_2_] treatment effects a one-way ANOVA was used to assess differences in variance between samples. A two-way ANOVA was used to assess species and [CO_2_] effects on *G*_smax_ (**Figure 3**). The relative change (Δ) in parameters was expressed as a percentage of the values recorded at 2000 ppm [CO_2_] relative to control values measured at 400 ppm [CO_2_]. Linear regression was used to investigate potential relationships between stomatal characteristics such as stomatal pore length and speed of stomatal closure and whether relative changes in stomatal behavior (i.e., Δ *G*_s_ change to [CO_2_] or darkness) were associated with the relative change in photosynthetic physiology.

## Results

**FIGURE 2:**
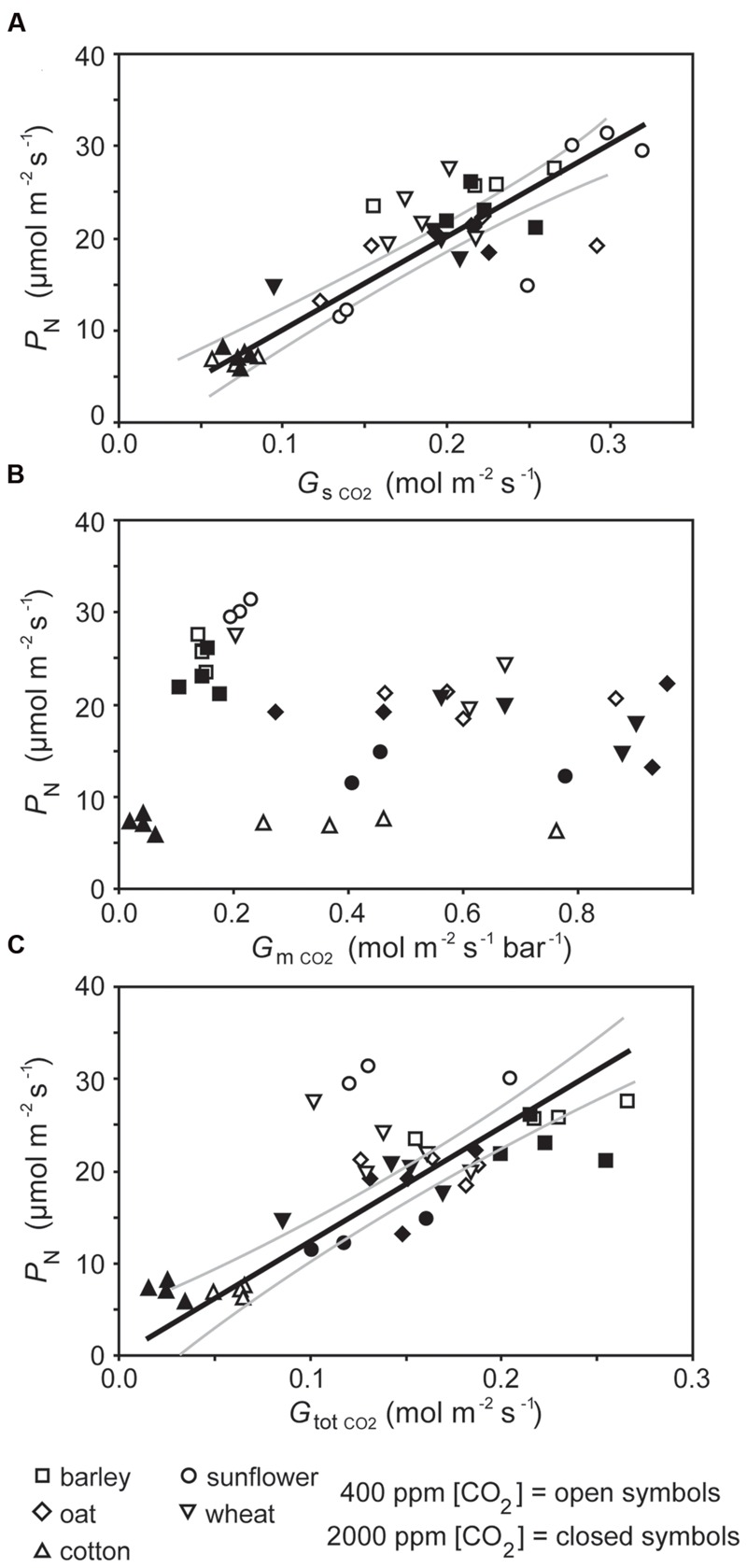
**The relationship between photosynthesis (*P*_N_) and **(A)** stomatal conductance to CO_2_ (*G*_sCO2_; linear regression *F*_1,37_ = 107.922; *P* = 1.607 × 10^-12^; *R*^2^ = 0.863), **(B)** mesophyll conductance to CO_2_ (*G*_mCO2_; linear regression *F*_1,37_ = 0.052; *P* = 0.821), and **(C)** total conductance to CO_2_ (*G*_totCO2_; linear regression *F*_1,37_ = 46.763; *P* = 4.659 × 10^-8^; *R*^2^ = 0.747).** Solid black line indicates best fit, gray lines either side indicate 95% confidence intervals of the mean.

**FIGURE 3:**
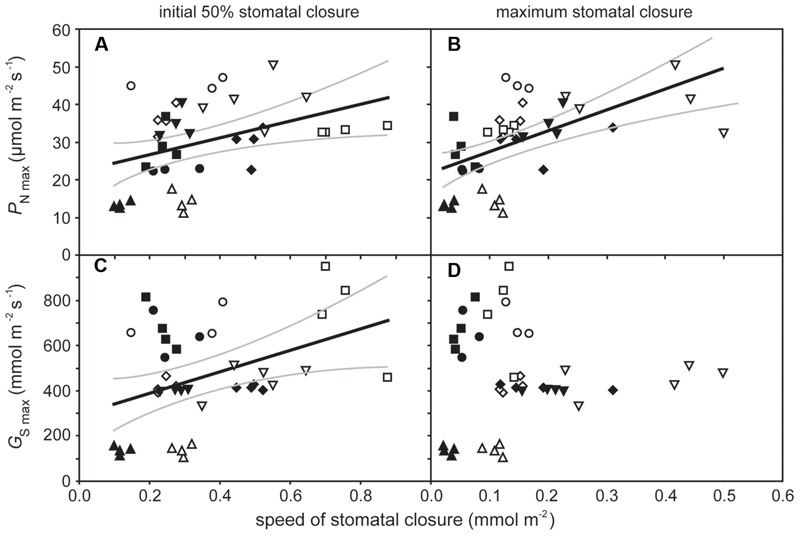
**The relationship between maximum rates of photosynthesis (*P*_N max_) and speed of stomatal closure during the initial 50% closure **(A)** (linear regression *F*_1,37_ = 6.814; *P* = 0.0130; *R*^2^ = 0.394) and to the maximum extent of stomatal closure, **(B)** (linear regression *F*_1,37_ = 17.654; *P* = 0.00016; *R*^2^ = 0.568); and the relationship between maximum rates of stomatal conductance (*G*_smax_) and speed of stomatal closure during the initial 50% closure, **(C)** (linear regression *F*_1,37_ = 7.235; *P* = 0.0107; *R*^2^ = 0.404) and to the maximum extent of stomatal closure, and **(D)** (linear regression *F*_1,37_ = 0.058; *P* = 0.811).** Solid black line indicates best fit, gray lines either side indicate 95% confidence intervals of the mean. Symbols as in **Figure 2**.

**FIGURE 4:**
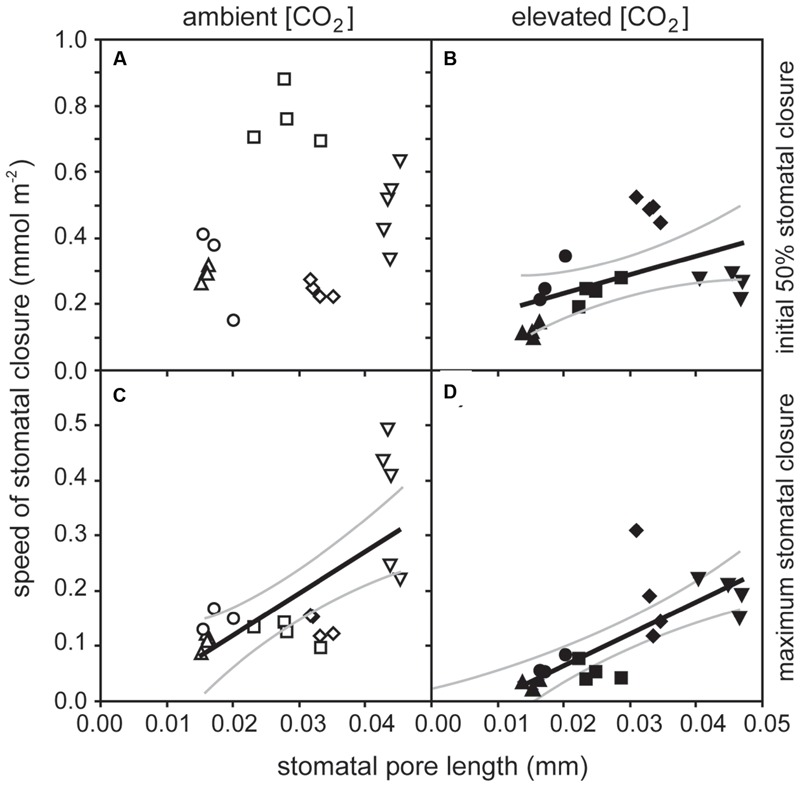
**The relationship between the stomatal pore length (SPL) and speed of stomatal closure to darkness: **(A)** speed of stomatal closure during the initial 50% of closure versus SPL of plants grown at 400 ppm [CO_2_] (linear regression *F*_1,37_ = 1.358; *P* = 0.259); **(B)** speed of stomatal closure during the initial 50% of closure versus SPL of plants grown at 2000 ppm [CO_2_] (linear regression *F*_1,37_ = 0.0331; *P* = 5.378; *R*^2^ = 0.490); **(C)** speed of stomatal closure during the time taken to achieve maximum closure versus SPL of plants grown at 400 ppm [CO_2_] (linear regression *F*_1,37_ = 16.304; *P* = 0.0008; *R*^2^ = 0.689), and **(D)** speed of stomatal closure during the time taken to achieve maximum closure versus SPL of plants grown at 2000 ppm [CO_2_] (linear regression *F*_1,37_ = 24.190; *P* = 0.0001; *R*^2^ = 0.766).** Solid black line indicates best fit, gray lines either side indicate 95% confidence intervals of the mean. Symbols as in **Figure 2**.

The rate of *P*_N_ in the five crop species grown in atmospheres of ambient and elevated [CO_2_] was positively related to *G*_sCO2_ and *G*_totCO2_ when measured at a common [CO_2_] level of 400 ppm (**Figure 2**). However, *P*_N_ did not correlate to *G*_mCO2_ measured using the curve fitting approach. Stomatal conductance to water vapor was 18.5–48.9% lower at elevated [CO_2_] in four of the species when measured at their respective growth [CO_2_] levels. Cotton showed no change in *G*_s H2O_ when grown at 400 and 2000 ppm [CO_2_] (**Table 1**; **Figure 2**). Positive relationships were observed between the speed of stomatal closure to darkness and *P*_N_ max (**Figures 3A,B**). The maximum rate of stomatal conductance was significantly correlated to the speed of stomatal closure during the initial 50% reduction in *G*_s H2O_ (**Figure 3C**), but not to the maximum extent of stomatal closure (**Figure 3D**). In plants grown at an ambient [CO_2_] of 400 ppm, no relationship was observed between SPL and the speed of stomatal closure during the initial 50% reduction in *G*_s H2O_ (**Figure 4A**). However, a positive relationship was observed between SPL and the speed of stomatal closure to the maximum extent of stomatal closure at ambient [CO_2_] (**Figure 4C**). Positive relationships were observed between the speed of stomatal closure and SPL in the five crop plants grown at elevated [CO_2_] (**Figures 4B,D**).

**Table 1:** The effect of growth at ambient (400 ppm) and elevated (2000 ppm) [CO_2_] on maximum rate of carboxylation of ribulose-1,5-bisphosphate carboxylase/oxygenase (*V*c_max_), the maximum rate of electron transport required for ribulose-1,5-bisphosphate regeneration (*J*_max_), stomatal conductance of water vapor (*G*_sH2O_) and chlorophyll fluorescence parameters of the maximum (*F*_v_/*F*_m_) and actual (ΦPSII) quantum efficiency of photosystem II.

Species	*V*c_max_ (μmol m^-2^ s^-1^)		*J*_max_ (μmol m^-2^ s^-1^)		*G*_s H2O_ (mmol m^-2^ s^-1^)		*F*_v_/*F*_m_		ΦPSII	
	Amb [CO_2_]	Elev [CO_2_]	Amb [CO_2_]	Elev [CO_2_]	Amb [CO_2_]	Elev [CO_2_]	Amb [CO_2_]	Elev [CO_2_]	Amb [CO_2_]	Elev [CO_2_]
Oat	87.8 ± 8.6^a^	70.7 ± 6.7^a^	144.6 ± 7.5^a^	123.5 ± 10.2^a^	669.7 ± 10.7^a^	439.4 ± 62.4^b^	0.842 ± 0.002^a^	0.858 ± 0.017^a^	0.427 ± 0.020^a^	0.436 ± 0.035^a^
Wheat	133.0 ± 4.0^a^	121.9 ± 6.8^a^	174.9 ± 11.1^a^	148.0 ± 8.8^a^	511.2 ± 26.5^a^	416.4 ± 57.0^a^	0.838 ± 0.004^a^	0.837 ± 0.003^a^	0.505 ± 0.034^a^	0.494 ± 0.015^a^
Cotton	68.4 ± 8.8^a^	34.2 ± 2.7^b^	73.5 ± 2.9^a^	75.1 ± 5.2^a^	138.3 ± 6.3^a^	137.7 ± 5.6^a^	0.827 ± 0.003^a^	0.823 ± 0.001^a^	0.233 ± 0.004^a^	0.338 ± 0.013^b^
Sunflower	123.4 ± 10.7^a^	61.1 ± 13.2^b^	173.3 ± 24.5^a^	96.9 ± 4.2^b^	976.5 ± 122.1^a^	498.6 ± 93.6^b^	0.854 ± 0.003^a^	0.858 ± 0.006^a^	0.566 ± 0.019^a^	0.560 ± 0.030^a^
Barley	72.4 ± 0.9^a^	66.9 ± 3.5^a^	137.0 ± 1.0^a^	120.8 ± 8.7^a^	874.0 ± 60.4^a^	585.5 ± 18.4^b^	0.845 ± 0.004^a^	0.840 ± 0.004^a^	0.438 ± 0.033^a^	0.397 ± 0.012^a^

*Values are the mean of four to six replicates. ± indicates standard error. Letters indicate significant difference determined with a one-way ANOVA.*

**FIGURE 5:**
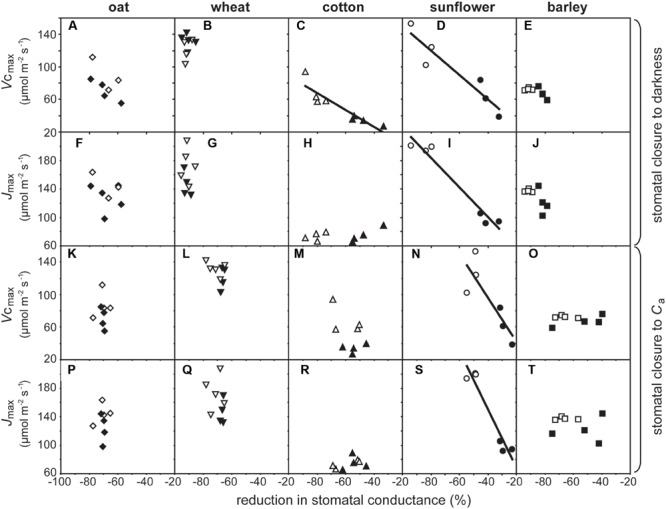
**The impact of growth at an elevated [CO_2_] of 2000 ppm on photosynthetic physiology and stomatal sensitivity to *C*_a_ and closure to darkness.** Linear regression was used to calculate *F* and *P* values are as follows: **(A)** *F*_1,6_ = 2.889, *P* = 0.140; **(B)** *F*_1,7_ = 0.079, *P* = 0.787; **(C)** *F*_1,6_ = 29.981, *P* = 0.00155, *R*^2^ = 0.913; **(D)** *F*_1,4_ = 28.643, *P* = 0.00587, *R*^2^ = 0.877; **(E)** *F*_1,6_ = 0.312, *P* = 0.597; **(F)** *F*_1,6_ = 0.726, *P* = 0.427; **(G)** *F*_1,7_ = 0.0252, *P* = 0.878; **(H)** *F*_1,6_ = 1.430, *P* = 0.277; **(I)** *F*_1,4_ = 81.006, *P* = 0.0008, *R*^2^ = 0.953; **(J)** *F*_1,6_ = 5.604, *P* = 0.0557; **(K)** *F*_1,6_ = 0.0325, *P* = 0.863; **(L)** *F*_1,7_ = 1.549, *P* = 0.253; **(M)** *F*_1,6_ = 1.600, *P* = 0.253; **(N)** *F*_1,4_ = 9.705, *P* = 0.0357, *R*^2^ = 0.708; **(O)** *F*_1,6_ = 0.312, *P* = 0.597; **(P)** *F*_1,6_ = 0.108, *P* = 0.754; **(Q)** *F*_1,7_ = 0.378, *P* = 0.558; **(R)** *F*_1,6_ = 1.309, *P* = 0.296; **(S)** *F*_1,4_ = 46.374, *P* = 0.00243, *R*^2^ = 0.921; **(T)** *F*_1,6_ = 0.184, *P* = 0.682. Symbols as in **Figure 2**.

**FIGURE 6:**
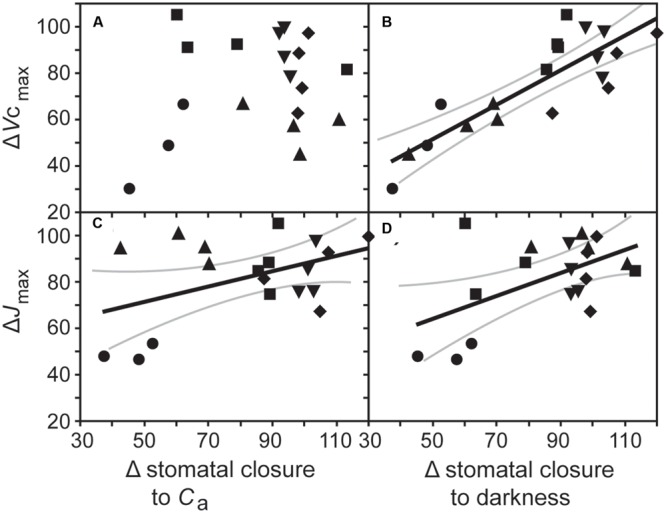
**The relationship between the proportional change in the maximum rate of carboxylation of ribulose-1,5-bisphosphate carboxylase/oxygenase (Δ*V*c_max_) with **(A)** the change in stomatal closure to a change in *C*_a_ from 400 to 2000 ppm [CO_2_] (Δstomatal closure to *C*_a_; linear regression *F*_1,17_ = 0.819; *P* = 0.378), and **(B)** stomatal closure to darkness in plants grown at an elevated [CO_2_] of 2000 ppm (Δstomatal closure to darkness; linear regression *F*_1,17_ = 44.454; *P* = 3.966 × 10^-6^; *R*^2^ = 0.851), and the relationship between the proportional change in the maximum rate of electron transport required for ribulose-1,5-bisphosphate regeneration (Δ*J*_max_) with **(C)** Δstomatal closure to *C*_a_ (linear regression *F*_1,17_ = 7.244; *P* = 0.0154; *R*^2^ = 0.547), and **(D)** Δstomatal closure to darkness (linear regression *F*_1,17_ = 4.506; *P* = 0.0488; *R*^2^ = 0.458).** Solid black line indicates best fit, gray lines either side indicate 95% confidence intervals of the mean. Symbols as in **Figure 2**.

Growth at elevated [CO_2_] resulted in significant declines in *V*c_max_ in cotton and sunflower (**Table 1**). This coincided with lower stomatal closure to darkness (**Figure 5**). Sunflower showed lower *V*c_max_ and *J*_max_ that coincided with impaired stomatal closure to darkness and stomatal sensitivity to *C*_a_; however, Δ*V*c_max_ in all five species did not correlate to Δstomatal closure to *C*_a_ (**Figure 6A**). Oat, wheat, and barley did not exhibit any significant correlations between photosynthetic physiology and stomatal control (**Figure 5**). However, when the relative change in photosynthetic parameters was compared to the relative change in stomatal control a highly significant positive correlation was observed between Δ*V*c_max_ and Δstomatal closure to darkness (**Figure 6B**). In effect, those species that retain *V*c_max_ (i.e., a Δ*V*c_max_ close to 100%) also tend to maintain stomatal closure (i.e., a Δstomatal closure to darkness close to 100%) at high [CO_2_]; while species that exhibit reduced *V*c_max_ at high [CO_2_] also show lower ability to close stomata in response to the cessation of illumination. Less robust positive correlations were also observed between Δ*J*_max_ with Δstomatal closure to [CO_2_] (**Figure 6C**) and Δstomatal closure to darkness (**Figure 6D**).

## Discussion

### Co-ordination of Photosynthesis and Stomatal Control

Photosynthesis of the crop plants grown at ambient and elevated [CO_2_] when measured at a common [CO_2_] was closely related to stomatal and total conductance to CO_2_ (**Figures 2A,C**). However, *G*_m CO2_ derived from the curve fitting method (Ethier and Livingston, 2004) did not show any significant relationship to *P*_N_ either at a common *C*_a_ (**Figure 2B**) or the respective growth [CO_2_] levels of the plants (data not shown). The curve fitting approach calculates a single *G*_m_ value along the *P*_N_ – *C*_i_ curve. The efficacy of this approach is reliant upon *G*_m_ remaining constant at a range of *C*_i_ values (Tazoe et al., 2009, 2011), however, measurement of *G*_m_ using the variable J method of Harley et al. (1992) across a *C*_i_ gradient suggests that rates of *G*_mCO2_ may not be uniform and instead vary with the availability of CO_2_ in relation to rates of *P*_N_ and respiration (Flexas et al., 2007). The curve fitting method has been successfully applied to the determination of *G*_mCO2_ in plants grown at identical [CO_2_] but different levels of water availability (Miyazawa et al., 2008). However, no relationship was observed between *P*_N_ and *G*_mCO2_ of the crop plants grown at different levels of [CO_2_] in the present study (**Figure 2B**). This may suggest that there is no effect of growth at elevated [CO_2_] on *G*_mCO2_ in the crop species analyzed as there were limited reductions in photosynthetic capacity of four of the five species due to the free availability of nutrients (Kitao et al., 2015), or a limitation in the effectiveness of the method due to the size of the cuvette employed (2 cm^2^; Pons et al., 2009). Movement of CO_2_ within the leaf is unlikely to have affected the *P*_N_ – *C*_i_ curves (Pons et al., 2009), as the species analyzed in this study possess heterobaric leaves (Nikolopoulos et al., 2002).

The speed of stomatal closure showed a positive relationship to *P*_N_._max_ and *G*_s_._max_ (**Figure 3**). This suggests that species with high potential rates of *P*_N_ and *G*_s_ require a high degree of stomatal control to reduce transpirative water-loss and prevent desiccation when conditions are not conducive to *P*_N_. This relationship may be indicative of natural selective pressures induced by declining [CO_2_] during the Cenozoic (65 Ma to present; Haworth et al., 2011) and artificial selective pressures during the domestication of crop species (Evans, 1980; Roche, 2015) favoring highly effective physiological stomatal control alongside high rates of *P*_N_ and *G*_s_. The trend of declining [CO_2_] during much of the past 65 million years (Berner, 2006) is considered to have favored species with large numbers of small stomata as the most effective arrangement of the epidermal surface to achieve maximum rates of gas exchange (Franks and Beerling, 2009; de Boer et al., 2016) in conjunction with rapid stomatal opening and closing (Hetherington and Woodward, 2003; Raven, 2014). In contrast to previous observations of a negative relationship between stomatal size and the speed of the adjustment in the size of stomatal aperture in closely related species with identical stomatal complex morphology (Drake et al., 2013), this study showed a positive relationship between stomatal size and the speed of the stomatal response to darkness (**Figure 4**). However, this positive relationship reflects the diversity in stomatal complex morphologies of the five plants studied. The dicots, cotton, and sunflower, both have stomatal complexes composed of ‘kidney-shaped’ guard cells; whereas the monocot grasses, oat, wheat, and barley, have larger ‘dumb-bell’ type guard cells (Haworth et al., 2015). The dumb-bell stomatal complexes of grasses may open and close more rapidly than kidney-shaped stomatal complexes. Stomatal opening relies upon an increase in guard cell turgor as ions are pumped across cell membranes to reduce water potential; however, to effectively open the larger dumb-bell guard cell pairs there needs to be a corresponding loss of turgor in the surrounding cell epidermal subsidiary cells to accommodate an increase in stomatal aperture (Franks and Farquhar, 2007). This rapid control and co-ordination of changes in cell turgor may account for the generally faster rates of stomatal closure observed in the grasses analyzed in this study (**Figure 4**), and ability of grasses to support larger stomatal pore apertures than species with kidney-shaped stomatal complexes.

### The Effect of Elevated [CO_2_] on Active Stomatal Behavior

Regulation of stomatal aperture size is achieved through a complex hierarchical sensory and signaling network (Hetherington and Woodward, 2003; Kim et al., 2010; Merilo et al., 2014). The results of this study (**Figure 6**) and others (e.g., Heath and Kerstiens, 1997; Heath, 1998; Zeppel et al., 2012) suggest that growth at elevated [CO_2_] may affect either the network of stomatal sensing/signaling or the physical function of stomata. Physiological stomatal control may occur via a signal from the mesophyll to the guard cells (Mott et al., 2008; Fujita et al., 2013), or as a result of metabolic changes within the guard cells (Talbott and Zeiger, 1998). It is not possible from the present dataset to definitively state whether the reduction in photosynthetic physiology and impaired stomatal function at high [CO_2_] are causally linked or co-incidental. Quantitative trait loci responsible for *P*_N_ and stomatal control occur within the same region of the genome in sunflower (Hervé et al., 2001), indicating the importance of their co-ordination in plant responses to environmental change. The mesophyll is the site where the majority of *P*_N_ occurs and mesophyll *P*_N_ and *G*_s_ are closely linked (Messinger et al., 2006). The results of this study may suggest that the effects on photosynthetic capacity and stomatal control at high [CO_2_] are associated. Growth at high [CO_2_] can cause damage (Madsen, 1971), inhibition (Van Oosten et al., 1994), and feedback limitations (Sage et al., 1989) to the photosynthetic physiology. However, the species studied in this experiment all exhibit fairly rapid growth that would reduce the impact of sink limitations (Manderscheid et al., 2010), and did not experience any limitations in nutrient availability that might promote any reduction of photosynthetic capacity at elevated [CO_2_] (Farage et al., 1998). Three of the five crops showed no effect of growth at elevated [CO_2_] on *P*_N_ – *C*_i_ curves, and none of the five crops exhibited lower *F*_v_/*F*_m_ or ΦPSII values at the higher [CO_2_] that might indicate some impairment or loss of performance of PSII corresponding to damage to the thylakoid membranes (Shaw et al., 2014; Kalaji et al., 2016) that may account for the patterns observed in *P*_N_ and stomatal control reported in this study.

An increase in [CO_2_] commonly induces a reduction in stomatal aperture in plants with active physiological stomatal control (Morison and Gifford, 1983; Haworth et al., 2013). Separation of the epidermis and mesophyll layers suggests that this is the result of a signal derived from the mesophyll such as sucrose or malate (Mott et al., 2008; Fujita et al., 2013) regulated by carbonic anhydrase within the mesophyll (Hu et al., 2010). An increase in transport of sucrose to the apoplastic space surrounding the guard cells may act as a signal to induce stomatal closure and limit water-loss during episodes where levels of photosynthetic sugar production exceed rates of transport from the photosynthetic organs (Outlaw, 2003). The plants grown at 2000 ppm [CO_2_] in this study exhibited stomatal sensitivity to an increase in *C*_a_ from 400 to 2000 ppm; however, the degree of closure in cotton and sunflower was lower, possibly indicating impairment of the mesophyll to guard cell signal, or physical damage to the stomata constraining the ability to close. The leaves of plants grown at high [CO_2_] frequently contain greater concentrations of soluble sugars due to CO_2_-fertilization and the accumulation of photosynthate if the capacity to transport sugars is exceeded (Van Oosten et al., 1994; Paul and Driscoll, 1997). Such an increase in soluble sugars in the apoplast at high [CO_2_] may impair the efficacy of a sucrose mesophyll derived signal, or the sensitivity of guard cells to apoplastic fluctuations in sucrose concentrations. The concentration of sugars such as sucrose derived from guard cell *P*_N_ may also play a role in maintaining guard cell turgor after the influx of potassium ions responsible for stomatal opening (Talbott and Zeiger, 1998). Disruption to carbohydrate metabolism may also influence guard and subsidiary cell osmotic balance, thus affecting the mechanics of stomatal opening/closing (e.g., Franks and Farquhar, 2007) and possibly accounting for impaired stomatal control in sunflower and cotton (**Figure 5**).

Stomatal opening in response to sub-ambient [CO_2_] occurs in isolated epidermal strips without the mesophyll, but stomatal closure induced by [CO_2_] levels above ambient requires chemical contact between the mesophyll and epidermal layers, suggesting the operation of a diffusible signal (Fujita et al., 2013). No difference was observed in *G*_smax_ values recorded at a *C*_a_ of 50 ppm [CO_2_] (two-way ANOVA: CO_2_, *F*_1,38_ = 1.580, *P* = 0.219; species *F*_4,38_ = 52.511, *P* = 7.160 × 10^-13^) of plants grown at 400 and 2000 ppm [CO_2_] (**Figure 3C**). However, *G*_s_ sensitivity to an instantaneous increase in [CO_2_] from 400 to 2000 ppm did alter between species; indicative of differential stomatal control mechanisms to sense and signal sub-ambient [CO_2_] (Fujita et al., 2013) and the photosynthetic control of [CO_2_] at higher levels of [CO_2_] (Messinger et al., 2006). The results of this study suggest that control of *G*_s_ by mesophyll *P*_N_ via *C*_i_ is influenced via the effect of growth at elevated [CO_2_] on photosynthetic physiology in cotton and sunflower with kidney-shaped stomatal complexes (**Figure 6**). The similarity in stomatal response to a sub-ambient *C*_a_ of 50 ppm, but difference in stomatal sensitivity to super-ambient *C*_a_ may indicate that growth at elevated [CO_2_] does not affect the physical aspects of stomatal opening/closing but rather disrupts the photosynthetic control of *G*_s_. In essence, the photosynthetic mesophyll to guard cell signaling mechanism appears to be operating in the same manner, but at high [CO_2_] the fine control of stomatal behavior is impaired. Stomatal aperture during *P*_N_ is linked to *C*_i_ (Roelfsema et al., 2002). Stomatal conductance in cocklebur (*Xanthium strumarium*) showed a gradual change when *P*_N_ was CO_2_-limited and a more dramatic reduction with *C*_i_ when *P*_N_ was limited by the regeneration of ribulose-1,5-bisphosphate (Messinger et al., 2006). This may account for the lack of correlation between Δ*V*c_max_ and stomatal sensitivity to [CO_2_] (**Figure 6A**) and the negative correlation between Δ*J*_max_ and Δstomatal closure to [CO_2_] (**Figure 6C**) observed in this study; suggesting that alteration of photosynthetic capacity may influence the photosynthetic determination of stomatal aperture in response to fluctuations in *C*_i_.

The pronounced reduction in the effectiveness of stomatal closure to darkness with *V*c_max_ (**Figure 6B**) may also be suggestive of a link between *P*_N_ and physiological stomatal behavior or impaired physical operation of the stomata. A diverse range of stomatal morphologies (Franks and Farquhar, 2007) and physiological behaviors (Haworth et al., 2013, 2015) are observed in evolutionarily diverse plants. The species utilized in this study all exhibit active physiological behavior (Haworth et al., 2015), but possess contrasting stomatal morphologies. The monocot grass species with dumb-bell guard cells generally retained the ability to close stomata, while the kidney-shaped stomata of the two dicots closed less effectively at high [CO_2_] (**Figure 6C**). Dumb-bell guard cells exhibit larger and faster changes in turgor than kidney-shaped guard cells (Franks and Farquhar, 2007). The guard cells of grasses do not possess chloroplasts (Brown and Johnson, 1962) and possibly reply upon the movement of potassium for stomatal opening (Fairley-Grenot and Assmann, 1992). To accommodate the rapid and large change in dumb-bell guard cell turgor, the surrounding subsidiary cells also undergo osmotic adjustment (Franks and Farquhar, 2007). This may suggest that the differential effect of elevated [CO_2_] on stomata of grasses and dicots found in this is due to differences in the biochemistry of stomatal opening related to the presence/absence of guard cell chloroplasts, stomatal signaling or photosynthetic control of stomatal aperture.

### Implications of Reduced Stomatal Control at High [CO_2_]

The results of this study suggest that one unexpected impact of elevated [CO_2_] may be a loss of stomatal function in some C3 herbaceous crop species. This impaired stomatal control corresponded to reduced carboxylation capacity, consistent with linkage between *G*_s_ and *P*_N_. Growth at elevated [CO_2_] generally reduced *G*_s H2O_ (**Table 1**). This lower *G*_s H2O_ was not associated with a decrease in stomatal density, but a reduction in stomatal aperture via physiological stomatal control (Haworth et al., 2015). Under normal growth conditions this lower *G*_s H2O_ will result in reduced water-loss and correspondingly greater WUE, consistent with observations of enhanced WUE in other experimental studies of elevated [CO_2_] (Centritto et al., 2002; Wullschleger et al., 2002; Ainsworth and Rogers, 2007). However, loss of stomatal function at elevated [CO_2_] may impair the ability of crop plants with active physiological stomatal behavior to exert stomatal control in the event of a change in growth conditions. Specifically, the reduced ability of stomata to close at elevated [CO_2_] may have significant implications for the capacity of crops to tolerate drought and heat stress in the future (e.g., Killi et al., 2016) and warranting further experimental investigation.

The aim of this study was to investigate the effects of elevated [CO_2_] on stomatal control by using a very high [CO_2_] level of 2000 ppm. This concentration is beyond the range of the IPCC worst case scenario (IPCC, 2007), but not above levels of [CO_2_] that have occurred over Earth history since the origination of vascular plants (e.g., Berner, 2009; Haworth et al., 2011). To investigate the loss of stomatal function under more realistic levels of [CO_2_] for the next 50–100 years further work should be undertaken in controlled environment chambers and free air CO_2_ enrichment (FACE) systems. However, many FACE systems do not increase [CO_2_] at night for economic reasons, instead operating enrichment only during daylight hours when *P*_N_ occurs. In terms of gauging the effect of elevated [CO_2_] on stomatal control it would be necessary for the plants to experience the most realistic simulation of future atmospheric conditions possible via continuous enrichment of [CO_2_] levels. The number and duration of heat-waves and drought events are predicted to increase in the future (e.g., Schär et al., 2004; Vautard et al., 2007). The capacity of crop plants to respond to and resist these adverse growth conditions is largely dependent upon effective stomatal control (Killi et al., 2016). At elevated [CO_2_], WUE may be higher and delay/mitigate the impact of drought (Wall, 2001; Wullschleger et al., 2002), but the results of our study and that of Heath and Kerstiens (1997) suggests that at severe drought the loss of stomatal function in C3 plants with kidney-shaped stomatal complexes could impair tolerance to drought. Further experimental work at [CO_2_] levels equivalent to those predicted in the next 100 years is required to assess whether the loss of stomatal control at high [CO_2_] may have negative implications for food security in a water-limited world.

## Author Contributions

MH and AR: Designed the experiments. MH, DK, and AM: Conducted the experiments. MH, DK, and MC: Processed data. MH, DK, AM, AR, and MC: Wrote the manuscript.

## Conflict of Interest Statement

The authors declare that the research was conducted in the absence of any commercial or financial relationships that could be construed as a potential conflict of interest.
